# Criminal Abortion across the Atlantic and on This Side

**Published:** 1899-01

**Authors:** 


### CRIMINAL ABORTION ACROSS THE ATLANTIC AND ON THIS SIDE.

This form of crime is very frequent in most parts of the world, and has been greatly in evidence of late in England, where, within the past six months, there have been tried three qualified medical men for performing an illegal operation with fatal results. One of these was sentenced to seven years penal servitude, while another has just been condemned to death. It is stated in a reliable English medical journal that the police have full knowledge of a ring of medical men in a certain quarter of London who regularly practice as abortionists, and that active steps are being taken to bring them to justice. In this country, although there has been no great public scandal for a considerable time, the fact that abortion prevails to a large extent is well known. Dr. Storer, of Newport, who has for many years been an active anti-abortionist, referring to the matter a few months ago, said: "Much has been written on the subject from various standpoints, but fetal murder still prevails, a dreadful monster that, wounded at one point, evinces but fresh strength at others. The decrease of the rate of increase still goes on." It may then be taken for granted that the practice is a common one. What are the reasons for this state of affairs, and what means will most effectually put a stop to its continuance. The reasons are as clear to read as an open book, and are simply that the well-to-do woman of the present day is, for the most part, averse to undertaking the duties and responsibilities of maternity, probably believing, too, in the majority of cases, that before the fourth month or thereabouts the fetus is but a part of its mother and is not endowed with separate life, and that, therefore, in procuring abortion, she is not guilty of any deadly sin. How to effectually prevent abortion is a most difficult problem to solve. With many women even the knowledge that in undergoing an illegal operation they were conniving at murder, would not act as a deterrent, while it would be difficult to convince many more that in procuring abortion they were committing a sin of any magnitude. The only course open would, therefore, seem to be to punish those who are detected in the crime with the utmost severity, the path that is now being pursued in England, although we agree with the following remarks of the *Medical Press:* "We trust, however, that the action of the authorities will not be limited to the arrest of medical men alone; the 'smart' women who seduce needy practitioners into relieving them of the troubles of maternity are equally guilty, and they should not be allowed to escape. If a few examples were made of these women it is quite possible that the work of the abortionist would soon undergo a serious diminution." Although the obstacles in the way of altogether preventing abortion are probably insuperable, yet some steps should be taken to check its widespread practice. Putting on one side all questions of morality and regarding the matter wholly from the standpoint of health, it must be confessed that abortion, conducted on a large scale, is certain to sap the vitality and to impede the progress of any nation.—*Pediatrics.*

